# Calibration of Small Grating Spectrometers from 166 to 600 cm^−1^

**DOI:** 10.6028/jres.066A.021

**Published:** 1962-06-01

**Authors:** L. R. Blaine, Earle K. Plyler, W. S. Benedict

## Abstract

In order to provide standards for the calibration of small grating spectrometers over the region from 166 to 600 cm^−1^, tracings of the spectrum of atmospheric water vapor are presented. The lines are identified and tabulated. Wavenumbers obtained from energy levels derived from the best available high-resolution spectra are given, together with an indication of their relative reliability. The best lines are believed accurate to ±0.03 cm^−1^.

## 1. Introduction

The Commission on Molecular Structure and Spectroscopy of the International Union of Pure and Applied Chemistry has recently published [l]^2^ a set of provisional wavenumbers for the calibration of infrared spectrometers. These tables include the spectral region from 600 to 4,000 cm^−1^. There are now available commercial instruments which operate to 200 cm^−1^ and there is a need for calibrating wavelengths in this region. No doubt it will require several years before completely adequate standards of high precision can be obtained for this region. The purpose of this report is to present wavenumbers, between 166 and 600 cm^−1^, of lines of atmospheric water vapor that may serve as preliminary provisional reference standards.

## 2. Method and Results

The rotational spectrum of H_2_O is very rich in lines and extends from 0.74 cm^−1^ to beyond 1,000 cm^−1^. Its greatest intensity is near 200 cm^−1^, such that evacuation of the instrument or flushing with a dry inert gas is required. The appearance of the spectrum depends upon the resolution of the spectrometer and the conditions of path length, relative humidity, temperature, and pressure. These factors also will affect the apparent wavelength of individual peaks, most strongly when weaker lines occur blended with the line principally responsible for the absorption, or when several components fall within the slit width. The provisional standards presented here are graded according to their invariance to such changes in condition, and are to be considered most reliable only for the reported conditions.

Theoretical analysis of the water-vapor spectrum as observed with high resolution, both in the region of the pure rotation spectrum and the vibration-rotation bands in the shorter infrared [2, 3, 4, 5, 6, 7] has led to a quite complete interpretation of the observed features, and to the determination from the spectra of energy levels of the ground vibrational state. From these levels positions of all transitions in the 166 to 600 cm^−1^ region, including many weak lines, may be calculated. It is believed that the positions thus calculated are more reliable than observations, since they depend upon the Ritz combination principle and upon a smoothing process, which averages the errors of individual measurements and individual observations. References 2 to 7 describe the procedure in more detail and provide numerous examples of how well the calculated line positions agree with observations throughout the spectrum.

The present standards are based on a new smoothed set of energy levels, which are in close agreement with earlier sets such as those of reference 6. The changes are based principally on new measurements by Rao [8] in the regions 280 to 550 cm^−1^ and 1,450 to 1,700 cm^−1^; by Izatt [9] in the region 450 to 690 cm^−1^; and in this laboratory from 1,580 to 2,200 cm^−1^. The levels and molecular constants derived therefrom will be presented elsewhere. A complete listing of all the pure rotation lines, including weaker ones with intensities down to 10^−5^ of the strongest has been prepared; this contains nearly 600 entries in the region covered here.

**Figure 1:**
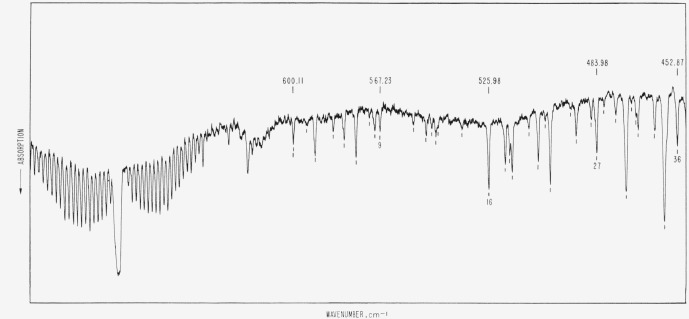
The atmospheric absorption from 680 to 450 cm^−1^ as observed on a small commercial grating instrument used single pass. The path length was 56 cm, the relative humidity 32 percent, and the temperature 68 °F. Lines included in table 1 are marked; a few are further identified by numbering as in table 1 below and by wavenumbers above the spectrum.

**Figure 2 f2-jresv66an3p223_a1b:**
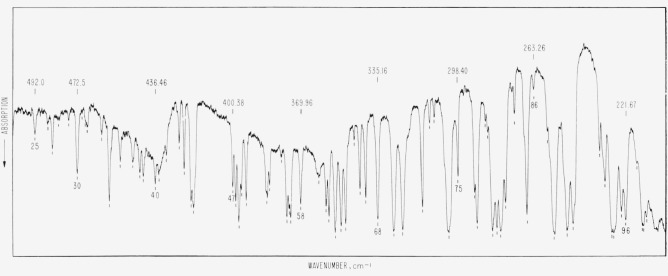
The atmospheric absorption from 500 to 225 cm^−1^. The experimental conditions were the same as for figure 1.

**Figure 3:**
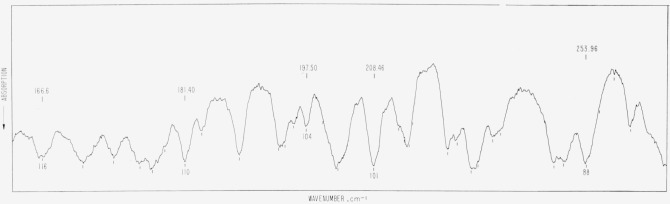
Atmospheric absorption from 225 to 160 cm^−1^ as observed on a small grating spectrometer. The path length was 65 cm, the relative humidity 32 percent and the temperature 68 °F.

Table 1 presents the recommended wavenumbers of lines, which are illustrated and numbered in figures 1 to 3. The entries are divided into three classes. Class A, the most reliable, is reserved for lines where a single transition or a very narrow (0.05 cm^−1^) doublet, is responsible for more than 90 percent of the absorption within 1 cm^−1^ of the line center. Class B lines are those where there are several minor components or wider but unresolved doublets, or where the line falls close to neighboring groups so that it is more dependent on conditions of observation. Class C lines are blends of several major components. It is believed that Class A lines should be reliable to better than ±0.03 cm^−1^; class B to ±0.1 cm^−1^; and class C to ±0.3 cm^−1^. [Fig f2-jresv66an3p223_a1b]

In the preparation of the illustrative figures, a commercial infrared grating spectrometer was used from 600 to 225 cm^−1^. The instrument was used single pass and the source compartment and the cell chamber were open to the air of the room, while the remaining part of the spectrometer was well dried. The effective slit was 0.4 cm^−1^, and the path length was 56 cm. The temperature and relative humidity are as specified in the captions. The carbon dioxide band is included in figure 1 in order to assist in the recognition of the water vapor lines. The identification and the wavenumbers of the lines of CO_2_ are given in another publication (see ref. 1).

The region from 225 to 166 cm^−1^ was measured on a small grating instrument with a resolution of about 1 cm^−1^, and the path length was 65 cm.

The absorption of the weaker lines in this region can be increased by the use of a longer path length or a higher relative humidity. The rotational lines shown in figures 1, 2, and 3 are for identification purposes only and the wavenumbers given in table 1 are based on measurements of higher resolution spectra.

## Figures and Tables

**Table 1:** *H_2_O* lines as calibration standards, 600—157 cm^−1^

Line No. and class	*v* cm^−1^	Line No. and class	*v* cm^−1^	Line No. and class	*v* cm^−1^
					
1 A	600.11	41 A	434.83	81 B	280.34
2 A	594.96	42 A	431.16	82 B	278.32
3 B	591.85	43 B	425.34	83 B	276.15
4 A	584.74	44 B	423.04	84 A	271.85
5 C	580.8	45 B	419.86	85 B	266.21
6 A	576.14	46 C	418.5	86 B	263.26
7 A	571.31	47 B	400.38	87 B	257.03
8 A	569.28	48 A	398.97	88 B	253.96
9 A	567.23	49 B	397.48	89 B	247.94
10 A	554.63	50 B	396.45	90 C	245.3
11 B	550.00	51 A	394.24	91 A	233.34
12 B	547.86	52 C	385.1	92 C	231.4
13 B	546.32	53 A	383.83	93 B	227.83
14 B	545.30	54 A	378.56	94 B	226.27
15 A	536.26	55 B	376.23	95 A	223.72
16 B	525.98	56 B	375.35	96 B	221.67
17 A	519.60	57 B	374.54	97 B	216.79
18 B	517.79	58 B	369.96	98 C	214.6
19 B	516.82	59 B	362.76	99 B	213.95
20 B	510.47	60 A	358.50	100 B	212.61
21 A	506.93	61 A	357.29	101 A	208.46
22 B	504.41	62 B	354.38	102 C	202.7
23 B	502.27	63 B	351.86	103 C	200.4
24 A	494.19	64 A	349.79	104 B	197.50
25 C	492.0	65 B	345.85	105 A	195.86
26 A	486.14	66 A	343.21	106 B	194.37
27 A	483.98	67 A	340.55	107 B	193.45
28 A	481.04	68 B	335.16	108 A	188.21
29 A	476.39	69 C	327.6	109 A	183.46
30 C	472.5	70 B	323.80	110 A	181.40
31 A	470.49	71 B	315.03	111 C	179.0
32 B	468.76	72 A	311.72	112 A	177.55
33 B	467.96	73 A	309.51	113 B	176.05
34 A	461.44	74 C	303.0	114 B	173.45
35 C	457.8	75 A	298.40	115 B	170.37
36 A	452.87	76 B	290.74	116 C	166.6
37 B	446.80	77 A	289.46	117 A	161.79
38 A	443.71	78 B	285.04	118 A	160.20
39 B	441.96	79 B	284.61	119 B	158.89
40 A	436.46	80 B	282 25	120 B	157.82

## Footnotes

2 Figures in brackets indicate the literature references at the end of this paper.
